# Investigation of Effect of Nutritional Drink on Chemotherapy-Induced Mucosal Injury and Tumor Growth in an Established Animal Model

**DOI:** 10.3390/nu5103948

**Published:** 2013-09-30

**Authors:** Emma Bateman, Joanne Bowen, Andrea Stringer, Bronwen Mayo, Erin Plews, Anthony Wignall, Norman Greenberg, Eduardo Schiffrin, Dorothy Keefe

**Affiliations:** 1 Mucositis Research Group, Centre for Personalised Cancer Medicine (CPCM), Centre for Clinical Research Excellence (CCRE) in Oral Health, Faculty of Health Sciences, University of Adelaide, Frome Road, Adelaide, SA 5005, Australia; E-Mails: joanne.bowen@adelaide.edu.au (J.B.); andrea.stringer@unisa.edu.au (A.S.); bronwen.mayo@health.sa.gov.au (B.M.); erin.plews@adelaide.edu.au (E.P.); anthony.wignall@adelaide.edu.au (A.W.); dorothy.keefe@health.sa.gov.au (D.K.); 2 School of Pharmacy & Medical Sciences, University of South Australia, City East Campus, Frome Road, Adelaide, SA 5005, Australia; 3 Nestlé Nutrition R&D Centers, 12500 Whitewater Drive, Minnetonka, MN 55343, USA; E-Mail: norman.greenberg@rd.nestle.com; 4 Nestlé Research Centre, Nestec Ltd., Vers-chez-les-Blanc, 1000 Lausanne 26, Switzerland; E-Mail: schiffrin@bluewin.ch; 5 RAH Cancer Centre, Royal Adelaide Hospital, Adelaide SA 5000, Australia

**Keywords:** mucositis, nutritional drinks, animal models

## Abstract

Chemotherapy-induced mucositis represents a significant burden to quality of life and healthcare costs, and may be improved through enhanced nutritional status. We first determined the safety of two nutritional drinks (plus placebo), and then potential gut protection in tumor-bearing rats in a model of methotrexate-induced mucositis. In study 1, animals were fed one of two test diets (or placebo or control chow pellets) for a total of 60 days and were monitored daily. All diets were found to be safe to administer. In study 2, after seven days of receiving diets, a Dark Agouti Mammary Adenocarcinoma (DAMA) was transplanted subcutaneously. Ten days after starting diets, animals had 2 mg/kg intramuscular methotrexate administered on two consecutive days; after this time, all animals were given soaked chow. Animals were monitored daily for changes in bodyweight, tumor burden and general health. Animals were killed 10, 12 and 16 days after initially starting diets, and tissues were collected at necropsy. In study 1, animals receiving diets had gained 0.8% and 10.8% of their starting bodyweight after 60 days, placebo animals 4.4%, and animals fed on standard chow had gained 15.1%. In study 2, there was no significant influence of test diet on bodyweight, organ weight, tumor burden or biochemical parameters. Only animals treated with MTX exhibited diarrhea, although animals receiving Diet A and Diet C showed a non-significant increase in incidence of diarrhea. Administration of these nutritional drinks did not improve symptoms of mucositis.

## 1. Introduction

Gastrointestinal (GI) mucositis induced by chemotherapeutic agents is often dose-limiting and can lead to cessation of treatment, which significantly impacts patient quality of life and survival, and also poses a significant burden to healthcare costs. Currently, the treatment for the symptoms of GI mucositis, such as diarrhea, nausea and weight loss, is supportive only. However, extensive research into the pathobiological mechanisms of mucositis, as well as effective interventions that prevent the onset of gut-related toxicity, are ongoing [1].

Improved nutritional status before and during chemotherapy has been suggested to improve symptoms of mucositis [2,3,4]. Nutritional drinks enriched with transforming growth factor β (TGF-β), free l-glutamine, short-chain fatty acids (SCFAs) and whey proteins have been studied in a number of animal models, and have largely demonstrated protection against chemotherapy-induced gut damage and weight loss [3,4,5,6,7]. The additional growth factors and nutrients not only provide excess energy sources for GI epithelium, which assists in protecting these cells against catabolic conditions, but also have more specific biological actions intended to ameliorate tissue damage associated with mucositis [8,9].

Mucositis is an inflammatory state, modulated by mucosal damage and subsequent expression of proinflammatory cytokines such as TNF-α and NF-κB, which further compound mucosal damage and ulceration [10]. Inhibition of these inflammatory responses may therefore prevent development of mucositis and its symptoms; it is for this reason that agents such as whey proteins and TGF-β are included within nutritional supplements designed to prevent and/or improve mucositis [3,11]. Whey proteins are a rich source of l-cysteine, a precursor for glutathione (GSH) synthesis [12,13,14], and in this regard, whey proteins contribute to antioxidant activity within the GI tissues, which helps to protect against mucositis.

Similarly, TGF-β, an immunomodulatory cytokine, is known to stimulate enterocyte proliferation [3] and reduce intestinal inflammation induced by chemotherapy. Conversely, TGF-β2 is known to have an inhibitive effect on stem cell proliferation within the intestine, which has been shown to protect gut epithelium both *in vitro* and *in vivo* from the toxic effects of chemotherapeutic drugs such as methotrexate [7]. TGF-β is known for pleiotropism and its involvement in mucositis is likely to be dependent upon specific environmental conditions [6].

It is known that cancer produces a state of glutamine deficiency [9], and it is for this reason that many nutritional supplements designed with cancer patients in mind contain free l-glutamine, which is a preferred substrate for enterocytes within the GI tract. l-Glutamine encourages protein synthesis, enterocyte proliferation and anti-inflammatory effects, all of which play a role in prevention of mucositis [15,16,17]. It has also been shown that supplemental glutamine (as an oral gavage) assists uptake of methotrexate into tumor tissue (in studies in rats), increasing tumoricidal effects, while decreasing MTX-associated side effects, such as gut toxicity [11].

The development of animal models to mimic the clinical setting of mucositis has been invaluable, and has been utilized to characterize the effects of several nutritional drinks. Studies investigating Clinutren Protect^®^ [4,5], Modulen^®^ [6], TGF-β [3,7], and l-glutamine [11,17,18] for prevention of methotrexate-induced gut damage have been previously undertaken with success. However, it is known that tumor burden is associated with significantly increased chemotherapy-induced gut toxicity [19]. As such, tumor-bearing animals were used as part of this current study, firstly to ensure that the nutritional supplements did not have positive growth effects on tumor tissue, and secondly, to better reflect the patient setting of chemotherapy-induced mucositis. Previous studies using these supplements have also failed to compare gastrointestinal effects with animals fed only on normal chow; it is for this reason that we used animals fed solely on standard chow as controls, in addition to a group fed a placebo of the nutritional supplement.

In this study, Nestlé Health Science S.A. (Lutry, Switzerland) provided two powdered nutritional drinks, Clinutren Protect^®^ and IMPACT Advanced Recovery^®^, as well as a powdered placebo, to investigate potential protective effects on the GI tract against methotrexate-induced mucositis in both tumor-naïve and tumor-bearing animals.

## 2. Methods and Materials

### 2.1. Diets

Three blinded test diets were supplied by Nestlé Health Science S.A. it was not until the conclusion of the study that unblinding occurred. During experiments, diets were referred to as Diet A, B or C. Diet A was later revealed to be Clinutren Protect^®^ (Table 1), which includes whey proteins and SCFAs, l-glutamine and TGF-β2. Diet B was a placebo (Table 1). Diet C was IMPACT Advanced Recovery^®^ (Table 1), which includes whey proteins (source of TGF-β), medium-chain triglycerides, arginine, nucleotides and *n*-3 polyunsaturated fatty acids. Control diet was standard rat chow pellets (Table 1).

**Table 1:** Composition of diets.

	Clinutren^®^ Protect (Diet A)	Placebo (Diet B)	Impact^®^ (Diet C)	Standard Chow
	Energy distribution			
Protein	25%	25%	22%	23%
Lipid	35%	25%	25%	12%
Carbohydrate	40%	50%	53%	65%
	g/100 g powder	g/100 g powder	g/100 g powder	g/100 g powder
*Protein*	*29.6*	*29.6*	*22.0*	*20*
Casein	12.5	21.75	-	*
Whey	9.25	-	16.9	*
TGF-β	0.009	-	0.0014	*
Glutamine	9.45	-	-	*
Arginine	-	-	5.10	*
Alanine	-	9.80	-	*
Nitrogen	5.25	4.95	4.38 ^1^	*
*Carbohydrate*	*47*	*47*	*54*	*59.4*
*Lipid*	*18.2*	*18.2*	*11*	*10.5*
Canola oil	5.7	5.7	-	*
Corn oil	2	2	2.0	*
Lecithin	0.3	0.3	-	*
Medium chain	9.1	9.1	3.0	1.31
Triglyceride	-	-	-	*
Fish oil	0.5	0.5	4.6	*
*Minerals*	*2.00*	*1.80*	*2.18*	*2.9*
Sodium, mg	220	210	434	180
Potassium, mg	560	520	543	820
Chloride, mg	245	190	486	*
Calcium, mg	300	200	324	800
Phosphorus, mg	200	210	292	700
Magnesium, mg	50	35	93	200
Manganese, μg	500	500	81	104
Selenium, μg	16.1	16.1	19	0.4
*Kcal/100 g*	*575*	*485*	*525*	*334*

^1^ About 0.1 g nitrogen/100 g contributed by yeast RNA. * Exact composition of standard chow in terms of proteins, lipids and minerals unavailable in some cases; However, it is known that it does not contain glutamine or TGF-β; Standard chow contains fatty acids from canola oil, corn oil and fish oil.

### 2.2. Study Design

Female Dark Agouti rats, weighing 150–180 g were used for all experiments and were randomized to experimental groups using a random number table. Growing (non-adult) animals were used so as to better chart weight increases or decreases with addition of the test diets. Younger animals are also more responsive to chemotherapy (MTX), and our established models of chemotherapy-induced mucositis focus on non-adult animals.

Appropriate animal ethics approval was obtained from SA Pathology Animal Ethics Committee, with experiments conducted in accordance with the National Health and Medical Research Council (Australia) Code of Practice for Animal Care in Research and Training (2004).

#### 2.2.1. Study 1: Safety and Tolerability of Test Diets

Animals were housed in groups of 4 under standard conditions of 12 h light/dark cycles, with *ad libitum* access to water and diets. Rats were maintained on Diet A, B or C (or control) for a total of 60 days. Bodyweight was measured and plotted daily. Food intake was measured by weighing diets before and after placement in the cages; daily intake per rat was calculated by averaging the total amount of diet eaten per day.

#### 2.2.2. Study 2: Effects of Diet upon Tumor Growth and MTX-Induced Injury

**Figure 1:**
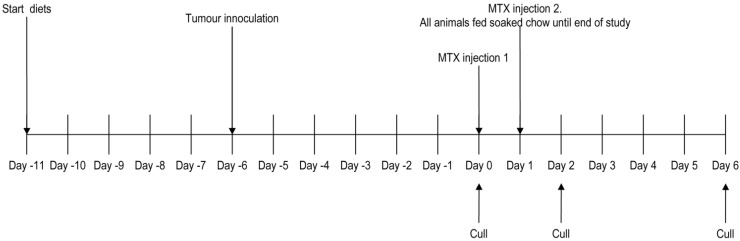
Experimental flow chart for study 2.

Animals were housed in groups of 6 under standard conditions of 12 h light/dark cycles, with *ad libitum* access to water and food. Half of each diet group (*n* = 6) was designated to the chemotherapy regimen; the remaining half was to receive saline injections only (*n* = 6). Test diets (or control) were administered from experimental day −9 to day 1 (11 days in total; Figure 1). On days 0 and 1, methotrexate or saline injection was administered (Figure 1). Thereafter, all animals were fed soaked standard chow to prevent diarrhea-induced dehydration and to ease alimentation until the end of the study (day 6; Figure 1). Food intake was monitored daily (as described above), as were bodyweights, tumor size, and general condition of the animals.

### 2.3. Tumor Inoculation

Animals had a suspension of Dark Agouti Mammary Adenocarcinoma (DAMA) cells (2 × 10^7^ cells/mL) implanted subcutaneously on each flank 6 days before beginning chemotherapy (experimental day −6, Figure 1). Tumors were measured with digital calipers as soon as they were palpable and tumor size and burden were calculated using the following formulae:

Tumor size = (Length × width × depth) × (π/6)
(1)

Tumor burden = [(right tumor size + left tumor size)/Bodyweight (g)] × 100
(2)

After tumor inoculation, animals were singly housed, and daily dietary intake was measured for individuals.

### 2.4. Chemotherapy

Methotrexate (2 mg/kg) or saline control were administered intramuscularly on experimental day 0 and day 1. At this time, tumor burden was calculated as approximately 3%–4% bodyweight. Animals were closely monitored daily. Those exhibiting severe diarrhea (grade 3 consistently for 24 h), severe weight loss (more than 15% of starting bodyweight), or a combination of symptoms signaling poor health (ruffled coat, hunched posture, reduced food and water intake, lowered body temperature) were euthanized under anesthesia via cervical dislocation, according to ethical requirements.

### 2.5. Necropsy

One third of all test groups were killed on experimental day 0 (*n* = 6), with another third killed on day 2 (*n* = 6), and the remaining animals were killed on day 6 (*n* = 6). Cardiac puncture was performed under anesthesia to collect blood and serum; these were processed for complete blood examination (CBE) and biochemical tests (MBA-20). Animals were then killed via cervical dislocation. Tumors were removed and weighed, and tumor wet weight was calculated as a percentage of bodyweight. Tumor tissue was then fixed in formalin for embedding in paraffin wax. Upon dissection, small and large intestines were removed and flushed with chilled saline, weighed, and sections of jejunum and colon were collected for tissue processing. Sections of jejunum (0.25 cm) were collected at approximately 20 cm from the gastro-duodenal junction, whereas 0.25 cm sections of colon were collected 1 cm down from the caecocolic junction.

### 2.6. Histochemical, Biochemical and Morphometric Assessment

All experiments were performed in a blinded fashion. Blood and serum were sent to SA Pathology for complete blood examination (CBE) and blood biochemistry tests (MBA-20). Formalin-fixed, paraffin-embedded colon and jejunum were cut into 4 μm sections and stained via a standard haematoxylin and eosin (H & E) protocol, or via alcian blue/periodic acid-Schiff mucin stain [20]. Sections were sent to a veterinary pathologist for examination. Immunohistochemistry to detect levels of caspase-3 (apoptosis marker) and ki67 (proliferation marker) was performed on 4 μm formalin-fixed, paraffin-embedded sections [21]. Microdissection to assess tissue morphometry (villous area and crypt depth) was performed by an experienced technician in our laboratory, as per established protocols [22].

### 2.7. Statistical Analyses

Data was analyzed using GraphPad Prism (version 5; GraphPad Software Inc., La Jolla, CA, USA). Where possible, two-way ANOVA was used, with Bonferroni’s *post hoc* test to determine group-specific differences; where data was non-parametric, or had missing values, the Kruskal-Wallis test was used, with Dunn’s *post hoc* test to determine differences between groups.

## 3. Results and Discussion

### 3.1. Study 1—Food Intake and Bodyweights

**Figure 2:**
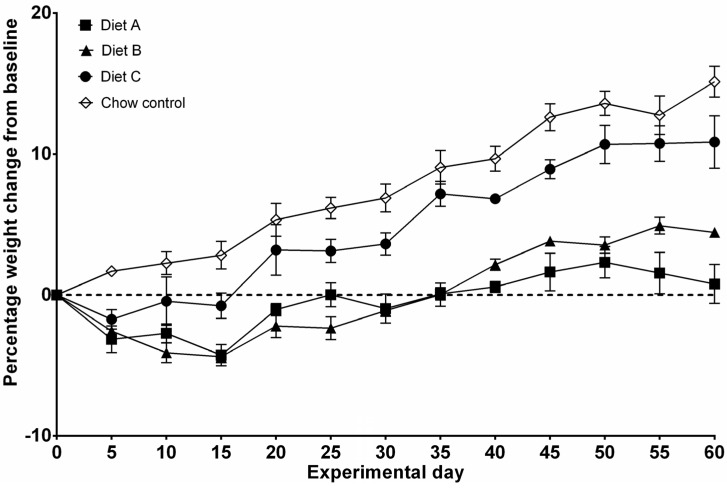
Percentage of weight change from baseline (study 1). Data are presented as mean ± SEM. Weight was measured daily, however, data points represent weigh every 5 days.

To assess safety of sole consumption of powdered test diets over a prolonged period, rats received Diet A, B or C, or standard chow for 60 days. Animals receiving test diets all lost weight in the first 24 h while adjusting to the change in diets (Figure 2). Diet C animals recovered this lost weight by day 13, Diet A animals recovered by day 25, and Diet B (placebo) animals recovered by day 35 (Figure 2). Chow control animals did not lose weight. After 60 days, animals receiving Diet A were 0.8% heavier, Diet B animals were 4.4% heavier, and Diet C animals were 10.8% heavier, compared to animals receiving standard chow, who gained 15.1% of their starting bodyweight (Figure 2). Diet-fed rats were significantly lighter than chow-fed rats over 60 days (*P* < 0.0001). In addition, animals fed on Diet A and B were significantly lighter than those fed on Diet C (*P* < 0.0001).

Rats receiving Diet C consumed the least initially, however, consumption increased by day 8, at which time, Diet C rats consumed more than animals receiving Diet A or B. Chow consumption was not quantified during this study, however, it was observed that chow-fed rats consumed more than their diet-fed counterparts. Consumption of chow pellets was quantified during study 2.

There were few significant differences in organ weights or blood biochemistry, except that animals receiving Diet A had significantly smaller livers than chow-fed rats (Kruskal-Wallis, *P* = 0.02), however, this significance was lost when corrected for bodyweight. There were some decreases in the levels of liver enzymes, particularly in animals fed on Diet A, however, no significance was reached.

Since there were no adverse effects due to administration of any of the test diets, it was considered safe to proceed with study 2.

### 3.2. Study 2

**Figure 3:**
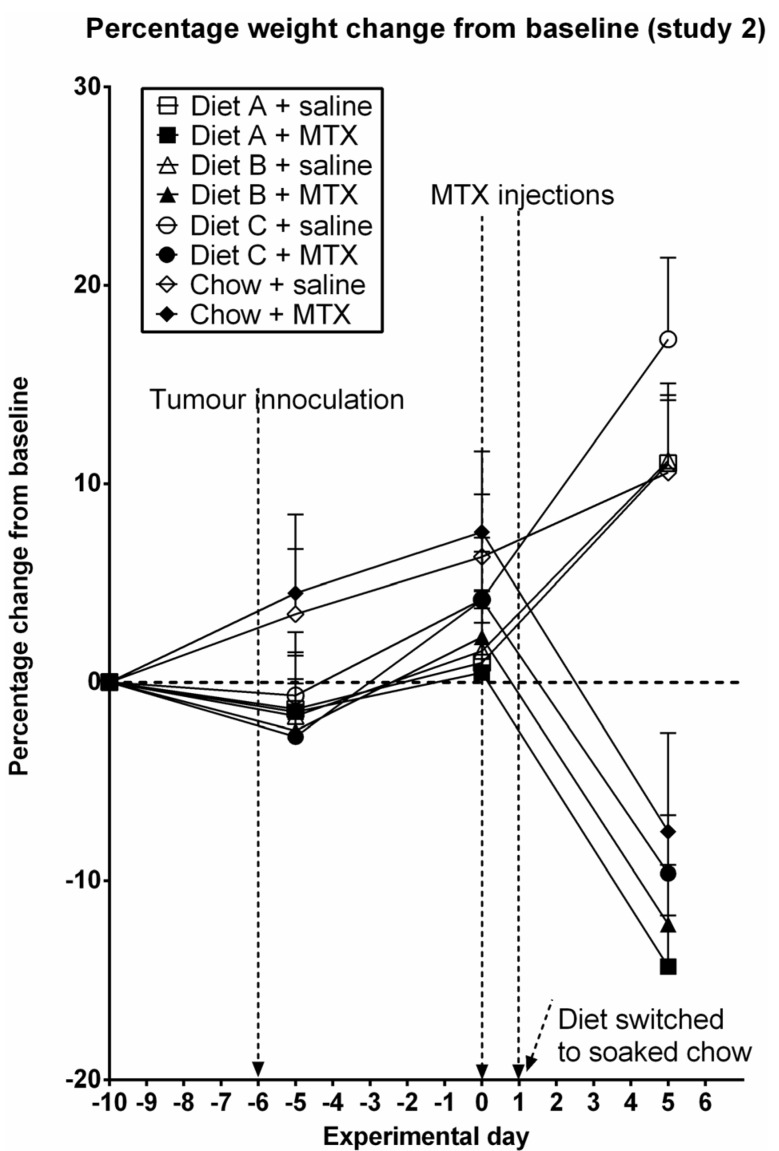
Percentage weight change from starting body weight (study 2) for each individual diet. Data are presented as mean + SEM. Weight was measured daily, however, data points represent weight every five days.

To assess the protective effect of test diets on mucositis in tumor-bearing rats, rats were given Diets A, B or C, or control up to the second MTX injection, from which time all animals were fed soaked standard chow (Figure 3). There was a non-significant decrease in weight in animals on test diets, which took 6–8 days to recover to pre-diet bodyweight (Figure 3). There was no significant effect of diet on bodyweight curves in saline-treated (*P* = 0.829) or MTX-treated (*P* = 0.935) rats (two-way repeated measures ANOVA, Figure 3), however, there was an increase in bodyweight in animals fed on Diet C compared to Diet A and Diet B (placebo) in both saline-treated and MTX-treated animals, however, this failed to reach significance (*P* = 0.935, Figure 3). Methotrexate treatment was associated with a significant decrease in bodyweight on days 4–6 (*P* < 0.01, Figure 3), with no significant differences between diet groups. Increased bodyweight in saline-treated animals compared to MTX-treated animals after experimental day 0 was likely to be due in part to increased tumor burden (Figure 3).

All animals treated with MTX significantly lost appetite regardless of diet, but appetite was recovering by day 6. There was no decrease in food consumption in saline-treated animals. Apart from a non-significant increase in food consumption in chow-fed animals up to MTX administration, there was no significant effect of diet on food consumption or MTX-related weight loss (two-way repeated measures ANOVA, *P* = 0.207).

### 3.3. Diarrhea

**Figure 4:**
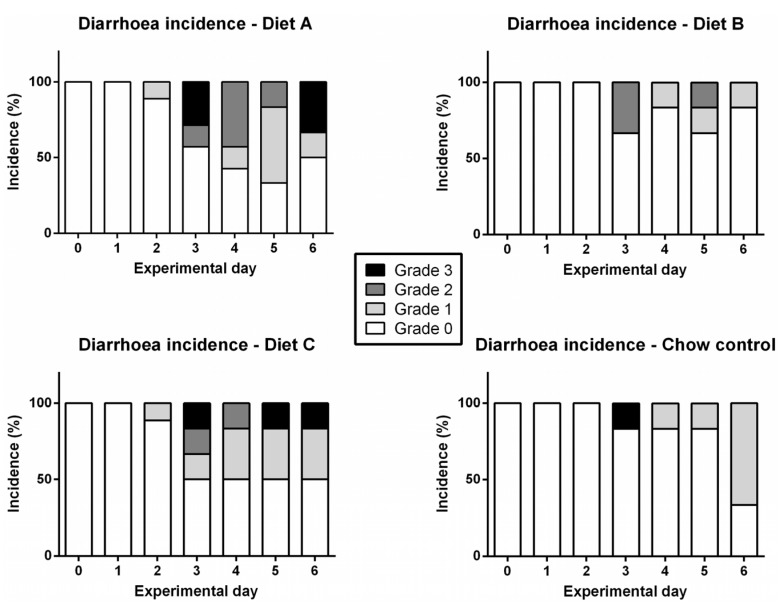
Incidence of diarrhea over six days in rats treated with chemotherapy (MTX).

No diarrhea was observed in study 1. In study 2, methotrexate treatment was associated with diarrhea, graded as 0 (none), 1 (mild), 2 (moderate) and 3 (severe) (Figure 4) [23]. No saline-treated animals experienced diarrhea. To determine whether diet had any effect on overall diarrhea incidence, a Kruskal-Wallis test was performed for rats treated with MTX; results failed to reach significance (*P* = 0.408), however, there was a non-significant increase in diarrhea incidence in rats fed on Diet A and Diet C, compared to control rats treated with MTX (Figure 4). Overall incidence of diarrhea (days 0–6) in Diet A-fed rats was 28.3%, and 28.8% in Diet C-fed rats, compared to 12.5% and 8.8% in placebo and control rats, respectively.

### 3.4. Tumor Growth

**Figure 5:**
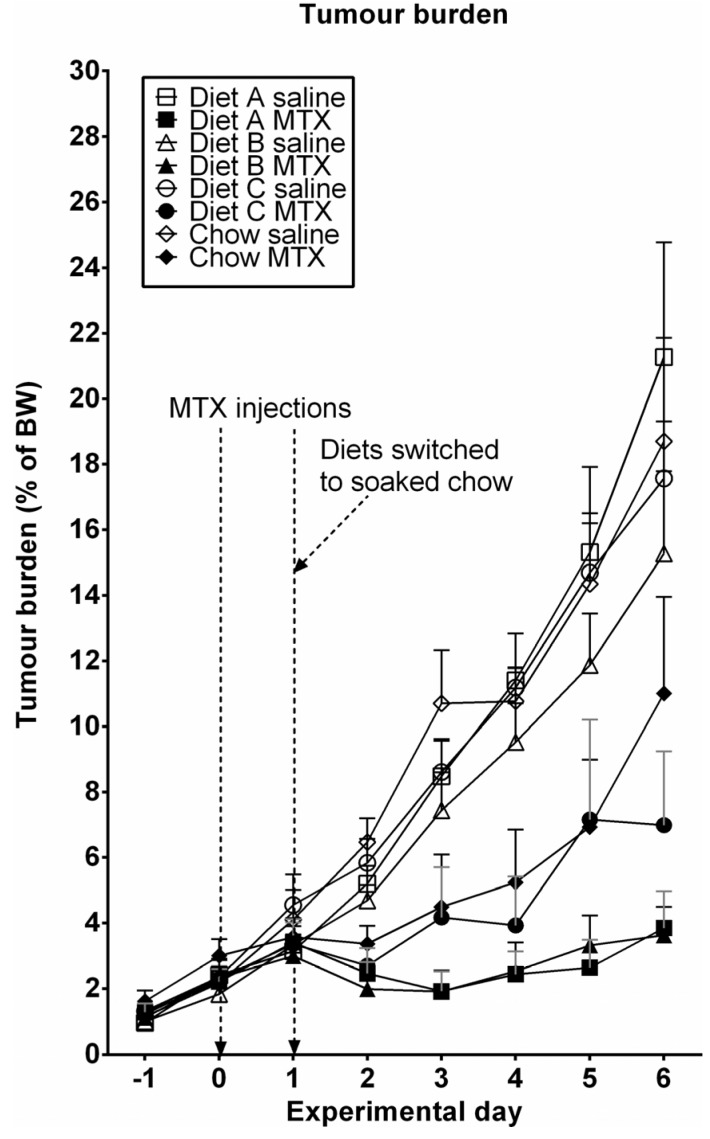
Tumor burden as a percentage of bodyweight. Data are presented as mean + SEM.

Tumors became palpable approximately 4–5 days post-transplantation, with tumors at day 0 (first day of chemotherapy) representing approximately 3%–4% of total body weight (Figure 5). MTX treatment resulted in decreased tumor burden in all groups, and there was a non-significant trend towards lighter tumors in rats fed on Diet A and placebo (*P* = 0.059) compared to Diet C and chow. Analysis of tumor wet weight as a percentage of bodyweight similarly revealed no significant effects of diet on tumor growth, in either saline-treated (*P* = 0.5572) or MTX-treated (*P* = 0.0676) animals.

### 3.5. Necropsy Data

Treatment with MTX was associated with significantly lighter spleens at day 6, irrespective of test diet (2-way ANOVA, *P* < 0.001), which is a common observation upon MTX administration. Small intestinal weight in MTX-treated rats was significantly decreased at day 2 and significantly increased at day 6 (2-way ANOVA, *P* < 0.0001) compared to saline controls. However, there was no influence of diet type on small intestinal weight, either as an absolute value or as a percentage of bodyweight.

The ingestion of non-food substances (pica) is an indirect marker of nausea in rats [24], which lack an emetogenic reflex. An increased amount of cage bedding was observed in the stomachs of MTX-treated animals, especially those fed on Diet A, which indicates nausea-induced pica. Analyses failed to detect any significant differences in stomach weights, however (2-way ANOVA, *P* = 0.2995). Of note, individual animals exhibiting pica tended to have higher grades of diarrhea (grade 2–3), compared to cagemates.

### 3.6. Complete Blood Examination and Blood Biochemistry

There were no significant changes in blood biochemistry markers. Methotrexate treatment was associated with a significant decrease in white blood cell counts at day 6 compared to saline treatment (*P* = 0.0447), however, this was not influenced by diet.

### 3.7. H & E and Tissue Morphometry

**Figure 6:**
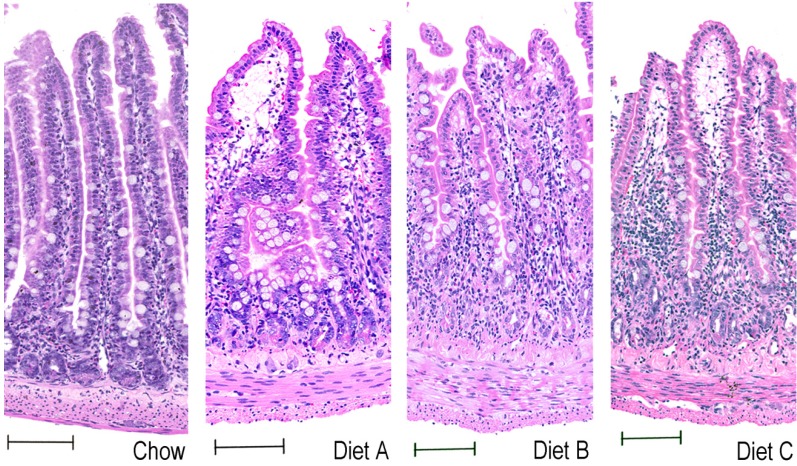
Representative photomicrographs of H&E-stained jejunum of MTX-treated animals (day 2), receiving Chow control, Diet A, Diet B and Diet C. Bars represent 100 μm. Original magnification 100×.

Examination of H & E-stained sections of jejunum and colon showed few significant changes to tissue architecture in response to test diets. Jejunum and colon of animals treated with MTX showed marked mucosal injury at day 2 (including crypt ablation, villous blunting, villous fusion and inflammation (Figure 6, jejunum), which had begun to resolve by day 6. Jejunum of MTX-treated animals also showed villous hyperplasia at day 6, which was reflected in significantly increased weight of small intestine at necropsy. Jejunum of animals receiving test diets had moderately increased lymphatic congestion in tips of villi (Figure 6), as well as increased inflammatory infiltrate throughout the mucosa (Figure 6). Villous blunting and fusion, as well as crypt disorganization and ablation were slightly more pronounced in jejunum of animals receiving test diets (Figure 6).

Analyses of villous height, area, crypt depth and mitotic figures via microdissection showed significantly increased mitotic figures and increased crypt depth in jejunum on day 6 (*P* = 0.0005 and *P* = 0.0003), and a non-signficant decrease in crypt depth and mitoses in colon at day 6 (*P* = 0.1470 and *P* = 0.4456) in tissues treated with MTX, however, no other significant effects were found, even when comparing test diets to chow.

### 3.8. Markers of Cell Proliferation (Ki67) and Apoptosis (Caspase-3)

Generally, Diet A, B and C rats had similar immunohistochemical profiles to chow rats in both MTX-treated and untreated sections. That is, few apoptotic cells were observed within untreated crypts with a small number of proliferating cells largely observed within the lower third of crypt bases (colon) and proliferating cells within the crypts and lower third of villi (jejunum). A non-significant decrease in the number of proliferating cells was detected for all MTX-treated sections on day 2 when compared to day 0, however the number of proliferating cells increased in day 6 MTX-treated sections. There were no significant differences detected between any of the diets or diet compared to chow.

Methotrexate treatment resulted in significant increases in the number of apoptotic cells (*P* = 0.035, Kruskal Wallis) when compared to untreated sections on days 2 and 6, particularly day 2, however, no significant differences were detected between any of the diets, or between the diets and chow.

### 3.9. Alcian Blue/Periodic Acid-Schiff Stain for Goblet Cells

This specialised stain was used to detect mucins within goblet cells of the jejunum and colon. It is known that cavitated cells are goblet cells that are extruding mucus via exocytosis. A higher number of cavitated cells suggest more mucosal damage [20,25]. There was a weakly significant increase in numbers of cavitated goblet cells in jejunum tissue of rats treated with MTX (at day 6, *P* = 0.03). However, multiple comparison testing could not determine significant differences between any of the diets.

### 3.10. Discussion

Due to the multifactorial nature of chemotherapy-induced mucositis, the protective effects of interventions are likewise multifactorial and dependent upon a number of biological parameters. Animal models of mucositis are extremely valuable in translational research, providing that care is taken to design the model such that it reflects the patient setting as closely as possible. We used a previously established animal model of methotrexate-induced mucositis to test three diets expected to ameliorate intestinal damage via multifactorial processes. Our study firstly determined the safety of administering test diets over several months, before secondly investigating the protective effects in a tumor-bearing model. Our study could not provide support for the use of the test diets for prevention of mucositis.

The test diets Clinutren Protect^®^ and IMPACT Advanced Recovery^®^ (and placebo) were found to cause altered weight gain compared to standard chow. In study 2, there was less clear separation between bodyweights due to diet as seen in study 1; this was most likely due to tumor burden, as this tumor model is known to cause some cachexia [26]. A plateau in food intake was observed in all tumor-bearing animals in the early stages of tumor growth, however, in saline-treated animals, both bodyweight and food intake increased after tumor burden had reached approximately 3%–4% of bodyweight.

Similar studies using Sprague-Dawley rats used three rounds of MTX chemotherapy, via subcutaneous injection of 2.5 mg/kg daily for three days [4,5]. It was shown that Clinutren Protect^®^ (CP) prevented fat loss in MTX-treated animals; these animals lost less weight overall than placebo-fed counterparts. However, incidence of MTX-induced diarrhea and mortality rates (53% in third round of chemotherapy) were not significantly improved in CP-fed animals. Although the treatment/rest cycles of MTX treatment in the study by Boukhettala *et al.* [4] mimic the patient setting more closely than the model used in the current study, tumor-naïve animals were used. Previous work by our group has shown that one cycle of two injections of 2 mg/kg MTX is sufficient to cause mucositis without causing excessive mortality, which correlates well with human clinical observations. Restricting the chemotherapy to one cycle allowed us to use tumor-bearing animals without excessively compromising animal health, while further mimicking the clinical setting. This is important, as it is known that tumor burden influences many parameters, such as toxicity [19]. Another limitation of the Boukhettala studies is that there were no chow-fed, or saline-treated controls. While animals fed on the CP diet fared slightly better than placebo-fed animals in terms of fat loss, the true measure of any positive effects of CP against weight loss or diarrhea were not apparent as a comparison against untreated controls.

Protective effects conferred by test diets may have been more prominent had they been administered as an oral gavage supplement to standard chow, or incorporated into chow pellets, rather than as the sole diet for test animals. The absence of an emetogenic reflex in rats means that other indicators of nausea and gut damage must be used, such as pica [24,27] (the ingestion of non-food items) and diarrhea. In the current study, rats administered 2 mg/kg MTX displayed signs of pica, namely, the ingestion of large amounts of cage bedding, which was found on necropsy. This was common in all animals fed powdered diets, but was most apparent in CP-fed animals, particularly those that exhibited severe diarrhea. This can be tied to toxicity clustering, but it seems that the lack of dietary roughage in diet-fed animals in this study heightened this phenomenon, and prevented any positive anti-inflammatory or growth-promoting effects of the nutritional drinks. This is unsurprising, as dietary bulk is required for normal gastrointestinal function in rats and humans [28], including production of trophic gut hormones and mucin secretion [29].

Studies by Harsha *et al.* [6] used standard chow pellets containing the nutritional supplement (Modulen^®^, Nestlé; 60% calorific intake of total pellet), both as a three day pre-treatment for chemotherapy (2.5 mg/kg MTX for three consecutive days), and at the time of MTX administration. Animals that received Modulen^®^ as a pre-treatment lost significantly less weight upon MTX administration than animals receiving concurrent Modulen^®^, or chow controls [6]. Similarly, studies by van’t Land *et al.* [7] used TGF-β2 as a supplement to standard chow and showed significant protective effects against MTX-induced weight loss, whereby MTX-treated animals receiving the supplement only lost 3% of their bodyweight, compared to controls which lost 10%. In the current study, animals on all test diets lost weight initially, consumed less per day than controls, and were significantly lighter at the end of both studies than chow-fed animals; presumably, this was because they were unfamiliar with the powdered food. Using the nutritional drinks as a gavaged supplement to normal chow might well have afforded more gastrointestinal protection against methotrexate-induced mucositis, as shown in the studies by Harsha *et al.* and van’t Land *et al.* [6,7].

## 4. Conclusions

To conclude, the nutritional drinks Clinutren Protect^®^ and IMPACT Advanced Recovery^®^ were safe to administer and did not have any growth-promoting effects on tumor tissue. However, no protection against methotrexate-induced mucositis was observed; this may due to the limitations of this study—namely, the lack of dietary bulk to aid digestion.

## Abbreviations

CBE: complete blood examination
CP: Clinutren Protect
DAMA: Dark Agouti Mammary Adenocarcinoma
GI: gastrointestinal
GSH: glutathione
H & E: haematoxylin and eosin
IMVS: Institute of Medical and Veterinary Sciences
MBA-20: medical biochemical analysis-20
MTX: methotrexate
NF-κB: nuclear factor κB
SCFA: short chain fatty acid
TGF: transforming growth factor
TNF-α: tumor necrosis factor α
